# Nutritional and microbiological dynamics in the preparation of prahoc fish paste

**DOI:** 10.1371/journal.pone.0321834

**Published:** 2025-04-24

**Authors:** Channmuny Thanh, Sylvie Avallone, Vincent Chochois, Caroline Douny, Kevin Bethune, Hasika Mith, Chanthol Peng, Adrien Servent, Ingrid Collombel

**Affiliations:** 1 QualiSud, Université de Montpellier, Avignon Université, CIRAD, Institut Agro, IRD, Université de la Réunion, Montpellier, France; 2 Research and Innovation Center, Institute of Technology of Cambodia, Phnom Penh, Cambodia; 3 Department of Food Sciences, Laboratory of Food Analysis, Faculty of Veterinary Medicine, Fundamental and Applied Research for Animals & Health (FARAH), Veterinary Public Health, University of Liège, Liège, Belgium; Lusofona University of Humanities and Technologies: Universidade Lusofona de Humanidades e Tecnologias, PORTUGAL

## Abstract

Prahoc is a traditional fermented fish widely consumed in Cambodia. Nevertheless, the processing and nutritional values of this daily-consumed product were poorly described. This study offers a detailed analysis of the biochemistry, nutritional composition, and microbiota during the six-month Prahoc incubation. Macronutrients (e.g. lipids, proteins) are rather well preserved during the preparation of the fish paste but the fatty acid and amino acid profiles are slightly modified at the end of the unit operation. Free amino acids increased, which facilitates the *in vitro* digestibility of the final paste, while beneficial fatty acids, such as eicosapentaenoic and docosahexaenoic acids, decreased. At the end of the process, the peroxide value was nearly five times greater than the limit set by the Codex Alimentarius (10 meq O_2_/kg). Biogenic amines, particularly cadaverine, were present but remained within acceptable limits. Metabarcoding analysis revealed that salt-tolerant bacteria dominated the fermentation process, while fungal activity was minimal. Lactic acid bacteria, such as *Vagococcus* and *Streptococcus*, were predominant before salt addition, while the fish pathogen *Aeromonas* established itself immediately after. *Clostridium* remained steady throughout, and *Lentibacillus* became dominant after six months. Food safety concerns related to biogenic amines, peroxides, and *Clostridium* highlight the need for establishing standard operational practices among national processors to mitigate food risks.

## Introduction

Globally, fish is an essential nutritional resource and plays a major role in employment [1]. In Cambodia, fish and fish-based products are the primary sources of animal protein, accounting for 37% of total protein intake and 76% of consumed animal proteins, respectively [1,2]. The Tonle Sap Lake (TSL) is the country’s main source of fish. The freshwater fish species it hosts are particularly rich in protein, with levels ranging from 17.6% to 20.1% [3,4]. However, fish availability fluctuates with the seasons, with the most productive harvesting period from September to March.

Given the limited availability of cold chain infrastructure at the national level, traditional processing and preservation techniques are employed to extend the shelf life of fish and ensure a continuous supply of protein throughout the year [5]. Approximately 60% of the total fish production is consumed fresh, while other forms of processing include fermentation (18%), salting and drying (13%), smoking (5%), fish sauce production (2%), and various derived products (2%) [6].

Fermentation is a traditional technique that has been used for centuries to extend the storage time of food. It has been employed to preserve a variety of foods produced from animals, fish, and plants [7]. This preservation method frequently employs locally sourced and seasonally available foods, hence mitigating transportation expenses and the environmental impact linked to food preservation [8]. In the case of natural fermentation, the process does not necessitate any external energy input. Fermentation has the potential to enhance the nutritional composition of food products through the increase of matrix digestibility and the bioavailability of specific micronutrients. Additionally, it has the capacity to mitigate some anti-nutritional influences [9].

Fermented fish and products derived from fermented fish are incorporated into the dietary traditions of numerous nations across the globe, especially in Asia [8]. Their widespread appeal can be attributed not only to their exceptional flavour, texture, and nutritional value, but also to the straightforwardness of their production process, which is frequently carried out using traditional methods. In Southeast-Asia, fermented fish and fermented fish-based-products can be consumed as primary course, as side dish, or as condiment [10]. Regrettably, the prior investigation about fermented fish has indicated that conventionally prepared salted or fermented goods are frequently linked to foodborne botulism [11].

Prahoc, a traditional Cambodian fermented fish, is derived from the fermentation of freshwater fish and salt in hermetic containers for a duration of up to six months or longer. This product plays a significant role in the Cambodian diet, with an estimated daily consumption of 18 grams per person as main dish, condiment or side dish [12]. The raw fish and salt combination, which is abundant in proteins and lipids, serves as a compelling medium for the proliferation of many non-aerobic halophilic and halotolerant microorganisms [6,13]. To date, the investigation of microbial diversity in Prahoc samples has required a preliminary step using plate-counting techniques. Those researches were carried out on samples of Prahoc from local markets. Lactic acid bacteria, specifically *Tetragenococcus*, along with a limited number of yeasts and potentially dangerous strains including *Bacillus cereus*, *Clostridium spp*., *Staphylococcus aureus*, *Escherichia coli*, *Pseudomonas spp*., and other members of the *Enterobacteriaceae* family were detected [11,14].

Fermented fish microbiota from other parts of Asia (such as Malaysia, Thailand and China) were analysed using culture-independent techniques. The lactic-acid and halophilic genera *Tetragenococcus* (mostly *Tetragenococcus muriaticus*), *Halanaerobium* (mostly *Halanaerobium fermentans*) and *Lactobacillus* (mostly *Lactobacillus renniniwas*, *Lactobacillus sakei*, *Lactobacillus pentosus*) appeared to often be the most prevalent ones. Other beneficial bacteria were detected in Malaysian fermented fishes including *Staphylococcus nepalensis*, *Weissella confusa*, and *Bifidobacterium bifidum* [15]. Whereas possible spoilage microbial genera such as *Xanthomonas*, *Acinetobacter*, *Pseudomonas* and *Psychrobacter* were detected in Chinese and Thai fermented fishes [16–18]. The halophilic genera *Lentibacillus, Vagococcus* and *Halomonas* were fairly abundant in Chinese and African fermented fishes [19]. Some recent reviews listed more deeply the type of microorganisms found in worldwide fermented fishes, their enzymatic activities and their links to flavour formation [10,20]. However, very limited information on Prahoc is available in these reviews.

The generation of biogenic amines (BAs) during the fish fermentation processes was reported frequently [11,19]. Histamine and tyramine levels can exceed the maximum levels recommended by the Codex Alimentarius, and certain strains of *Enterobacteriaceae* are thought to be involved in the production of these compounds. On the contrary, an isolated strain of *Tetragenococcus halophilus* was found to have a suppressive effect on histamine production [21]. According to Abré et al. [19], *Lentibacillus* would bring a decrease of the major BAs when *Staphylococcus*, *Lactobacillus*, *Psychrobacter*, *Peptostreptococcus* and *Fusobacterium* would lead to an increase of these biogenic compounds.

The aim of our research was to address the knowledge gap regarding the mechanisms involved in the preparation of fish paste (Prahoc) using a traditional homemade recipe with *Channa micropeltes* fish. Molecular tools were employed to analyse the change in microbiota composition at different stages of fish paste preparation, alongside other analytical methods, such as the measurement of biogenic amines and *in vitro* digestibility.

## Materials and methods

### Chemicals and reagents

Solvents, reagents, and pure standards (fatty acid methyl esters standards) were obtained from Sigma-Aldrich (Saint Quentin Fallavier, France) and polytetrafluoroethylene (PTFE) membranes from Sartorius (Palaiseau, France). A commercial mixture of 17 amino acids (AAS18 standard) was obtained from Sigma-Aldrich (Chemie GmbH, Munich, Germany).

### Fish sampling

Ten kilograms of *Channa micropeltes* were purchased from the aquaculture producers located in Kampong Phluk Commune (Siem Reap Province, Cambodia). Fish were placed in a bag under ice in expanded boxes and transported to the laboratory. The fish paste was processed 6 h after collection. In parallel, two commercial products from two different producers from Siem Reap province were bought in the Kampong Phluk market. The sample MK1 was made of *Trichopodus microlepis* while the sample MK2 was made of *Channa micropeltes*. The fermentation of the two market samples lasted between 6 and 8 months at ambient temperature (25 to 30 °C). After sampling, they were placed in a bag and stored at −20 ± 2 °C until the analysis.

### Fish paste processing

The processing of the fish paste took place at the laboratory of the Institute of Technology of Cambodia (Phnom Penh). The traditional homemade recipe was described during a prior survey which was done in Siem Reap Province with the local processors. Briefly, this recipe was based on seven operation units (cleaning, filleting, soaking, pressing, salting, fermenting and grinding). The fish was first cleaned and filleted into small pieces excluding the head, bones and scales (raw material - RM). The chopped fish was then soaked at room temperature in water:fish (3:1, w/w) for a duration of 20 h (soaked fish - SF). The fish that had been soaked was pressed using traditional stone plates for a duration of 6 h in order to eliminate any extra water. Afterward, the fish was salted with NaCl 30% (Month 0). The paste was divided into two different batches, with each batch consisting of four polyethylene terephthalate (PET) bottles and contained 400 g of fish paste. They were incubated over a six-month-period at 30 ± 2 °C (FIRLABO AC60, France). Every two months, one sample from each batch was collected and thereafter stored at a temperature of −20 ± 2 °C for subsequent analysis (Month 2, Month 4 and Month 6). The physicochemical parameters and contaminants of the samples were assessed after defrosting. During fermentation, a liquid fraction separated from the fish flesh. As local consumers only eat the paste, it was separated from the liquid fraction before being grounded and analyzed.

### Determination of pH

pH values were measured on two grams of sample mixed with 20 mL of distilled water and left at room temperature for five minutes [22]. Measurements were then done using a pH meter (Hanna, pH 213, Italy).

### Determination of proximate composition

The dry matter content was determined by drying three g of fresh samples in a thermostated oven (Memmert, UF B 500, Germany) at 105 °C for 24 h. Water activity (aw) was measured by using a water activity analyzer (AQUALAB 4TE, Decagon Devices Inc., Washington, USA).

The total lipid content was determined as previously described with slight modifications [3]. Briefly, 1.5 g of dried sample was hydrated with 10 mL of distilled water for 10 min and then dispersed in 30 mL of chloroform/methanol (2:1, v/v) for 2 min at 10 000 rpm (Ultra-Turrax T8, IKA, Germany). The mixture was sonicated for 5 min (Ultrasonic bath, Fisher Scientific, Germany), magnetically stirred (Heidolph, Reax 2, overhead shaker, Germany) for 1 h at room temperature and centrifuged at 2500 × g for 30 min at 4 °C (Avanti J-E, Beckman Coulter, France). The upper layer was discarded and the lower one was rinsed with 0.9% NaCl and centrifuged again at 400 × g for 30 min at 4 °C. The organic layer was collected and evaporated at 40 °C (Genevac LTD, EZ-2 series, Sp Scientific, England). The total lipids (corresponding to the dry residue) were weighed.

Total nitrogen content was determined by Dumas’s method with an element analyzer (FP528-LECO Trumac N, EVISA, Europe) [23]. The crude protein content was calculated using a conversion factor of 6.25.

### Determination of fatty acid profiles

The fatty acids (FA) were esterified into methyl esters by acid catalysed methylation [24]. Briefly, 2 mL of sodium methoxide/methanol (0.8% w/v) was added to extracted lipids. The mixture was heated at 80 °C for 15 min, cooled down and neutralised with sulfuric acid 1M (phenolphthalein as colour indicator). Afterwards, the mixture was heated again at 80 °C for 5 min, and 4 mL of saturated chloride solution was added accordingly. Fatty acid methyl esters (FAME) were extracted in 1 mL of hexane and analysed by gas chromatography coupled with flame ionisation detection (Agilent 6890N, Agilent Technologies, Santa Clara, CA, USA) with a DB-WAX-Agilent column (100 m × 0.25 mm × 0.2 μm film, Sigma-Aldrich). FAME were identified according to their respective retention times compared with analytical standards.

### Determination of total amino acids

Total amino acids (TAA) were extracted from 15 mg of lyophilized samples and hydrolyzed by 450 µL methanesulfonic acid 4 N [25]. After addition of 50 µL norleucine as an internal standard, each hydrolysate was degassed with nitrogen and heated at 150 °C for 2 h (Reacti-Therm Heating and Stirring Modules, Thermo Scientific, USA). The reaction was stopped by 450 µL of NaOH 4 N. The mixture was transferred to a 5 ml vial and the volume made up to 5 mL with sodium loading buffer (pH = 2.2). The extracts were filtered with a 0.45 μm cellulose acetate membrane (Minisart NML, France). The amino acids (AA) were separated, identified and quantified by the Biochrom 30 Amino Acid Analyzer (Biochrom 30+, Biochrom Ltd., Cambridge, UK). Chromatography was performed on an ion exchange resin (sodium system) eluted with a series of buffers over the pH range of 3.2 to 6.4. Peak detection was achieved by mixing elute with ninhydrin at 135 °C and measuring the absorbance at 570 and 440 nm. Amino acids were identified by retention times and quantified by external calibration with pure standards. Calibration curves were built for compounds and contents were expressed in g/100 g of wet weight.

### Determination of free-amino acids

Free amino acids (FAA) were extracted from 200 mg of lyophilized samples by 4.95 mL of sodium loading buffer (pH = 2.2) with the addition of 50 µL norleucine as an internal standard. The solution was vortexed and magnetically stirred (Heidolph, Reax 2, overhead shaker, Germany) for 1 h at room temperature. After that, the solution was centrifuged at 2500 × g for 10 min at 4 °C (Avanti J-E, Beckman Coulter, France). The extracts were filtered with a 0.45 μm cellulose acetate membrane (Minisart NML, France) and injected into the Biochrom 30 Amino Acid Analyzer (Biochrom 30+, Biochrom Ltd., Cambridge, UK) as described in the previous section.

### Determination of salt content

Salt content of fish pastes was determined by using a chloride analyzer (Model 926 Chloride Analyser, Sherwood Scientific, UK). Five grams of sample were mixed with 45 mL of distilled water, and the measurements were carried out on the liquid fraction accordingly.

### Determination of organic acids

The organic acids (OA) were extracted from 100 mg lyophilized samples with 1.5 mL of H_2_SO_4_ 2 mM. The mixtures were vortexed, sonicated (Ultrasonic bath, Fisher Scientific, Germany) for 15 min and then magnetically stirred (Heidolph, Reax 2, overhead shaker, Germany) for 2 h at room temperature. After being centrifuged at 2500 × g for 10 min at 4 °C (Avanti J-E, Beckman Coulter, France), the supernatants were filtered with a 0.45 μm cellulose acetate membrane (Minisart NML, France).

The OA (acetic, butyric, citric, formic, lactic, malic, propionic, and tartaric acid) were separated by high-pressure ion chromatography (Thermo Scientific, Dionex ICS-5000+, USA) equipped with an anionic exchange column (Dionex Ion Pac, AS11-HC, 4 mm × 250 mm, Thermo Scientific, USA) at 30 °C. The eluent used was NaOH, whose concentration varied from 1.25 mM to 55 mM over 50 min using a generator (Dionex ICS 5000 EG, Thermo Scientific). Injection volume was 10 μL and detection was based on pulsed amperometry.

### Determination of peroxide value

Lyophilized samples (0.5 g) were mixed with 25 mL of acetic acid:chloroform (3:2, v/v) [26]. Then, 1 mL of saturated potassium iodide was added and the solution was shaken for 1 min. After 10 min in the dark, 30 mL of distilled water and 1 mL of freshly prepared 1% starch were added. The released iodine was titrated with sodium thiosulfate 0.01 N until the blue colour disappeared. The peroxide value (PV) was calculated as meq O_2_/kg of sample according to the following formula:


$$PV_{\left(mEq/kg\right)}=\left(Vs-Vb\right)\times N\times 1000/W$$

where, Vs is the volume of Na_2_S_2_O_3_ used for the fish sample (mL), Vb is the volume of Na_2_S_2_O_3_ used in the blank sample (mL), N is the normality of Na_2_S_2_O_3_ used for titration (mEq/ mL), and W is the weight of the sample (g).

### Determination of biogenic amines

The content in BA was assessed as previously described [27]. Briefly, 400 mg of lyophilized samples were spiked with 1,7-diaminoheptane as internal standard and extracted with 5 mL perchloric acid 0.4 mol/L. The extract was filtered through an Acrodisc® filter and pH of the solution was increased with NaOH 2 mol/L and saturated NaHCO_3_ before derivation with dansyl chloride (10 mg/mL in acetone) at 70 °C. After dansylation, glycine (150 mg/mL in water) was added to bind the excess dansyl chloride. Dansylated amines were analysed on a UPLC Acquity system integrated autosampler (Acquity Sample Manager FTN), solvent delivery system (Acquity QSM H Class) and column heater coupled to an Acquity Fluorescence detector, all from Waters Corporation. The column used was an Acquity UPLC BEH C18 (2.1 × 100 mm, 1.7 μm), with a UPLC BEH C18 VanGuard pre-column (2.1 × 5 mm, 1.7 μm), both from Waters Corporation. The elution was performed using water and acetonitrile at a flow rate 0.4 mL/min, with a total run time of 26 min. The oven temperature was set at 65 °C and the injection volume was 5 μL. Fluorimetric detection was performed at 346 nm for excitation and 500 nm for emission. The limit of quantification ranged between 0.8 and 10 mg/kg depending on the compound.

Finally, the biogenic amines index (BAI) was determined for each sample by summing the quantities of putrescine, cadaverine, tyramine and histamine [28].

### Dry matter digestibility using *in vitro* digestion

A static *in vitro* digestion model was used to assess the digestibility of dry matter from raw and fermented fish as previously described [29]. The stock solutions were prepared before the experiments while the enzymatic solutions were prepared extemporaneously. About 4.0 g of fish was weighed and 1 mL of distilled water was added. Then, 5 mL of salivary solution was added, and the sample was incubated for 3 min at 37 °C with gentle stirring. Then, 10 mL of gastric phase was added, and samples were incubated for 2 h at 37 °C with gentle stirring. The solution was adjusted to pH 5.0 with NaOH 1 M and then 20 mL of intestinal solution was added. The sample was incubated for 2 h at 37 °C with gentle stirring. After that, the liquid phases were separated from the solid residues by centrifugation at 10 000×g for 30 min at 15 °C (Avanti™ J-E, Beckman Coulter ®). To estimate the dry matter digestibility, 20 mL of the digesta were dried at 105 °C until constant weight.

### Culture-dependent analyses

At the end of fermentation (Month 6), 10 g of each sample was aseptically collected in a stomacher bag and homogenised for three minutes at 230 rpm in 90 mL of buffered peptone water (Stomacher 400 Circulator, Seward, UK). Enumeration of possible pathogens was carried out with a sample (0.1 mL or 1 mL) of minimum three decimal dilutions on various selective media by spread plate technique method or inoculation into the mass.

To detect specific microorganisms, several selective media were used and incubated under the following conditions:

**Total coliforms:** plate method on Violet Red Bile Lactose Agar (Biokar Diagnostics, France) incubated at 37 °C for 24 h.

**Sulfite-reducing *Clostridium perfringens*:** Tryptone Sulfite Neomycin Agar (Biokar Diagnostics, France) anaerobically incubated at 46 °C for 24 h.

***Bacillus cereus* group:** Bacillus Cereus Agar plus Polymyxin B and Egg Yolk Emulsion (Biokar Diagnostics, France) incubated at 37 °C for 24–48 h.

***Staphylococcus aureus*:** Mannitol Salt Agar (Biokar Diagnostics, France), incubated at 37 °C for 24–48 h.

The plates were screened for the presence of colonies after the incubation period and the numbers of bacteria were expressed as Colony Forming Unit (CFU)/g.

### Metabarcoding analyses

#### DNA extraction.

For each fish sample, three distant core sampling using a sterilised metal ring were mixed together. DNA was extracted three times (except for the market samples extracted only once) from approximately 200 mg of the mix by using DNeasy PowerSoil Pro Kit (QIAGEN, Germany). The extracts were quantified using the Qubit fluorometer (Invitrogen™). The total DNA concentrations were adjusted to 5 ng/µL for samples higher than 5 ng/µL. The others ranged from 0.58 ng/µL to 4.8 ng/µL.

#### DNA amplification and sequencing.

Variable regions ITS2 and 16S V3-V4, from fungal and bacterial rRNA genes were then amplified using two primer pairs, respectively ITS-86F/ ITS-4R [30] and 16S-341F/ 16S-785R [31]. PCR reaction mixtures contained 1 µL of DNA, 0.25 µM forward primer, 0.25 µM reverse primer and 10 µL of 2X Phusion Flash High Fidelity PCR Master Mix (Fisher Scientific™, ref. F548L) in a final volume of 20 µL. The final concentration of DNA in PCR mixture was 0.25 ng/µL for adjusted samples or less for lower concentration DNA extracts. Peptide nucleic acids clamps designed to suppress respectively chloroplast (pPNA) and mitochondrial (mPNA) contamination were added to the PCR reaction mixture for the 16S V3-V4 region at the final concentration of 0.25 µM (PNA Bio Inc, Newbury Park, CA, USA). PCR amplification for ITS2 region was carried out in duplicates in a thermocycler as follow: initial denaturation step at 98 °C for 5 min, followed by 30 cycles at 98 °C for 15 sec, 55 °C for 20 sec and 72 °C for 20 sec, with a final extension step of 72 °C for 5 min. While PCR amplification for 16S V3-V4 region was carried out in duplicates in a thermocycler as follow: initial denaturation step at 98 °C for 5 min, followed by 25 cycles at 98 °C for 15 sec, 78 °C for 5 sec, 54 °C for 20 sec and 72 °C for 20 sec, with a final extension step of 72 °C for 5 min. Amplicon size and intensity were checked on 2% agarose 1X TAE gel. PCR duplicates were pooled together and sent to the GenSeq platform of the University of Montpellier (France). The amplicons DNA were first purified by using magnetic bead purification (Clean PCR, Proteigene, Saint-Marcel, France), and a second amplification was performed to add Illumina indexed barcode sequences. PCR products were purified a second time and pooled together. The final library was paired-end sequenced on an Illumina MiSeq sequencer with a 2 × 300 bp MiSeq Reagent Kit v3 (600 cycles; Illumina, San Diego, CA, USA) aiming for a minimum depth of 20 000X.

### Statistical analysis

The proximate composition and physico-chemical parameters are presented as mean ± standard deviation for two samples analysed in duplicate at each time. The microbiological study is expressed as mean ± standard deviation for two samples analysed in triplication. Statistical analysis was performed using one-way analysis of variance (one-way ANOVA) using Excel. Significance was accepted at probability *P*<0.05. Comparison of means was performed using the Tukey test.

Sequencing data were demultiplexed and trimmed using cutadapt v4.0. Forward and reverse paired-end reads were then merged, cleaned from chimeras, filtered and assigned a taxonomy using a dada2 (v1.30.0) based workflow [32]. The taxonomy assignment databases used were silva 16S v138.1 and UNITE v9.0 databases for 16S V3-V4 and ITS2, respectively. Beta diversity population structure and composition were analysed using the phyloseq (version 1.46.0) [33] and microViz (version 0.12.4) [34] packages in R (version 4.4.1) after rarefaction to an even number of reads of 10 401 and 2 387 per sample for 16S and ITS2 respectively. Beta diversity differences between sampling steps were tested using permutational ANOVA (PERMANOVA, adonis2 function) with 99999 permutations.

## Results and discussion

### pH, salt content and proximate composition of fish paste at four times of controlled fermentation and from the market

The pH, salt content and proximate compositions of the fish paste were monitored over the six-month-period of storage (Table 1). During the course of the fermentation, the pH values were rather stable (from 6.5 to 6.2) and the end value is in agreement with a previous study [14].

**Table 1:** pH, salt content and proximate composition of fish paste at four times of controlled fermentation and from the market (n = 4).

Sample	pH	Dry Matter (%)	Aw	NaCl (%)	Lipids (g/100g)	Proteins (g/100g)
Month 0	6.5 ± 0.0^*^	35.0 ± 1.3^****^	0.81 ± 0.0^*^	15.1 ± 0.8^***^	2.7 ± 0.4^*^	17.2 ± 0.3^***^
Month 2	6.2 ± 0.0^***^	53.4 ± 3.2^*^	0.74 ± 0.0^**^	30.9 ± 3.4^*^	2.7 ± 0.1^*^	19.3 ± 0.2^**^
Month 4	6.2 ± 0.0^***^	49.5 ± 1.0^**^	0.74 ± 0.0^**^	26.6 ± 1.0^**^	2.6 ± 0.3^*^	20.2 ± 0.6^*^
Month 6	6.4 ± 0.0^**,#^	45.2 ± 3.1^***,#^	0.74 ± 0.0^**,#^	25.3 ± 2.0^**,#^	2.4 ± 0.3^*,#^	16.4 ± 0.3^****,#^
Market	6.4 ± 0.1^#^	48.1 ± 1.4^#^	0.74 ± 0.0^#^	21.4 ± 1.1^,##^	2.1 ± 0.1^#^	16.1 ± 0.5^#^

Results are expressed as mean ± standard deviation on the basis of wet weight. A different number of stars in the same column indicates a significant difference (P < 0.05) by Tukey test between different fermentation times in the laboratory. A different number of symbols (#) in the same column indicates a significant difference (P < 0.05) by Tukey test between laboratory and market samples.

The aw of the fish paste decreases significantly from 0.82 to 0.74 during the first two months of fermentation. This final value is close to that previously reported by other teams on fermented fish [11,14,22]. The diffusion of salt into pieces of fish flesh decreases the awand increases their dry matter from 35.0 to 45.2% [35]. It is generally accepted that an intermediate aw (between 0.6 and 0.7) inhibits the growth of pathogens and spoilage microorganisms [36]. In Southeast Asia, fermented fish often has a salt content of over 20% and can be classified as highly salted products [21]. Since the NaCl percentage was higher in the laboratory samples compared to the market sample, we can hypothesize that the producer may have used a sodium content below 30% in processing this market sample. Regular sodium consumption contributes to high blood pressure and is a major risk factor for cardiovascular diseases, potentially leading to strokes. In the long term, high sodium intake can also increase the risk of kidney disease and osteoporosis.

A six-month incubation period had no significant effect on the lipid content of the fish paste but resulted in a noticeable decrease in protein content, with a final reduction of 1.1%. Various authors have reported that during fermentation, a portion of the proteins is hydrolyzed by endogenous and/or microbial proteases, releasing peptides and amino acids [37]. Overall, the final products from the laboratory and the market showed very similar characteristics for most of the parameters studied indicating that our preparation of fermented fish in the laboratory is a good method to mimic that used traditionally to supply local markets.

### Organic acid profiles of fish paste at four times of controlled fermentation

**Fig 1:**
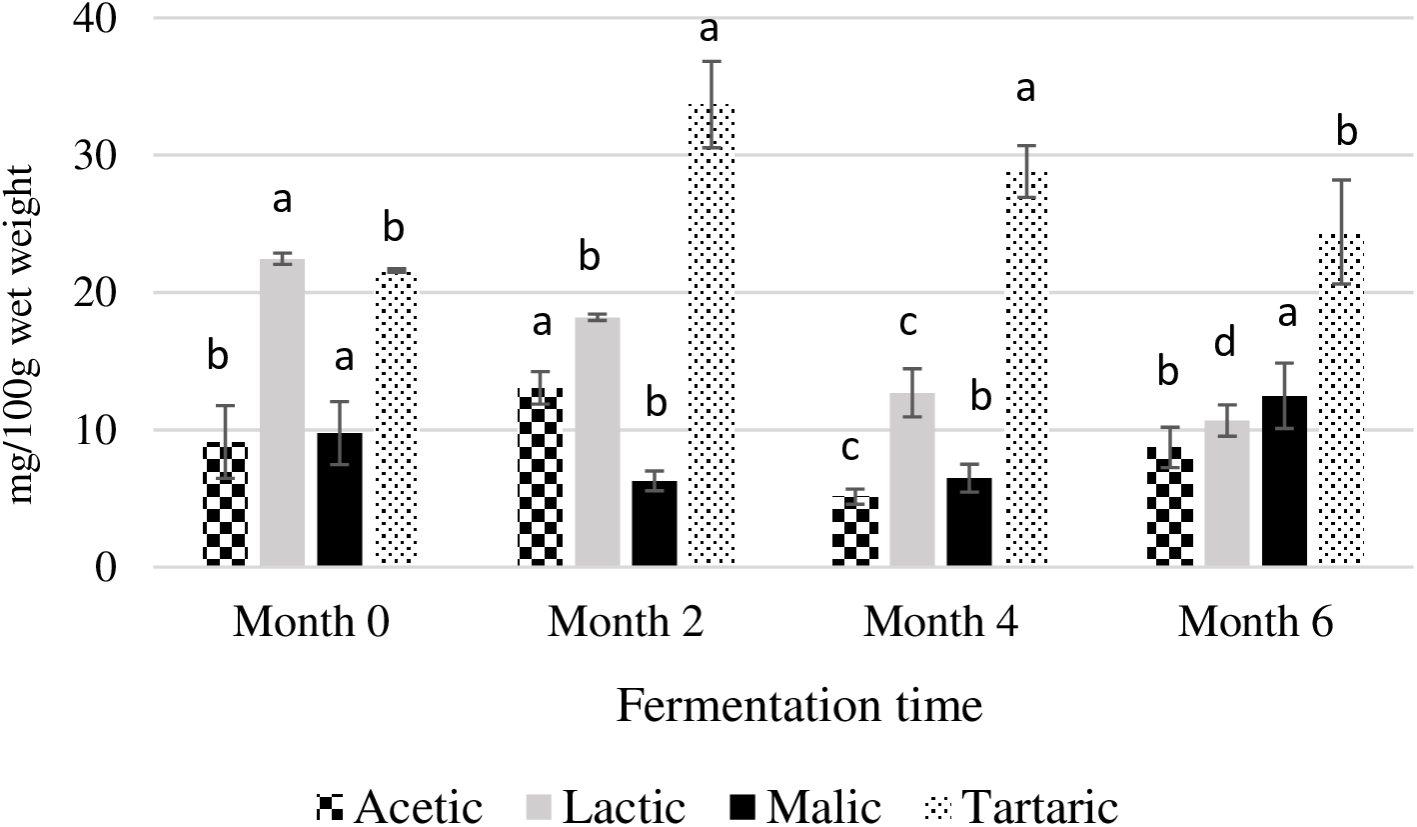
Organic acid profile of fish paste at four times of controlled fermentation (n = 4). Results are expressed as mean ± standard deviation in mg per 100 g of wet weight. Different letters for the same type of bar means a significant difference (*P* < 0.05) by Tukey’s test between different times.

Since the beginning of the fermentation, four OA were detected: acetic acid, lactic acid, malic acid and tartaric acid (Fig 1). When comparing the initial and final fermentation times, three organic acids remain stable, while lactic acid decreases significantly from 22.5 to 10.7 mg/100 g wet weight.

OAs are major flavours in traditional fermented foods, which can be produced by microorganisms [38]. The OA contents are influenced by many factors, including the environment, the kind of microorganisms present, and the raw material properties [39]. The fluctuation of organic contents was also found in a previous study on shrimp and fish paste fermentation [14,40].

### Fatty acid profiles of fish paste at four times of controlled fermentation

At each stage of the fish paste preparation, the main fatty acids were palmitic acid (C16:0), stearic acid (C18:0), behenic acid (C22:0) and oleic acid (C18:1) (Table 2). The saturated fatty acids (SFA) were the most abundant, followed by monounsaturated fatty acids (MUFA), and polyunsaturated fatty acids (PUFA) as previously reported for freshwater fish [3,41].

**Table 2:** Fatty acid profiles of fish paste at four times of controlled fermentation (n = 4).

Fatty acids	Month 0	Month 2	Month 4	Month 6
C14:0 (Myristic)	1.4 ± 0.7^*^	1.7 ± 0.7^*^	2.1 ± 0.3^*^	1.9 ± 0.2^*^
C16:0 (Palmitic)	19.3 ± 5.3^*^	20.3 ± 1.4^*^	21.8 ± 1.4^*^	19.7 ± 0.7^*^
C17:0 (Heptadecanoic)	6.5 ± 1.8^*^	6.0 ± 0.3^*^	6.6 ± 0.9^*^	6.1 ± 0.2^*^
C18:0 (Stearic)	17.3 ± 4.2^*,**^	18.8 ± 2.3^*^	13.6 ± 1.6^**^	13.8 ± 1.1^**^
C21:0 (Henicosanoic)	3.5 ± 1.1^*^	3.1 ± 0.3^*,**^	2.2 ± 0.5^**^	2.5 ± 0.5^*,**^
C22:0 (Behenic)	10.1 ± 4.9^*^	11.3 ± 0.3^*^	12.3 ± 0.9^*^	13.8 ± 1.3^*^
C24:0 (Lignoceric)	1.6 ± 0.3^*^	1.6 ± 0.1^*^	1.3 ± 0.1^*^	1.3 ± 0.2^*^
C16:1 (Palmitoleic)	5.1 ± 1.2^*^	5.0 ± 0.5^*^	5.6 ± 0.3^*^	5.1 ± 0.4^*^
C18:1n9c (Oleic)	11.8 ± 1.4^**^	11.7 ± 1.0^**^	14.4 ± 0.9^*^	13.0 ± 0.3^*,**^
C18:1n9t (Elaidic)	3.2 ± 0.2^*^	3.0 ± 0.2^*,**^	3.1 ± 0.5^*,**^	2.7 ± 0.1^**^
C22:1n9 (Erucic)	1.7 ± 0.9^*^	2.0 ± 0.4^*^	1.6 ± 0.5^*^	2.4 ± 0.1^*^
C18:2n6c (Linoleic)	0.8 ± 0.1^***^	1.1 ± 0.1^***^	2.2 ± 0.3^*^	1.7 ± 0.3^**^
C20:4n6 (Arachidonic)	0.8 ± 0.0^***^	1.1 ± 0.1^**,***^	1.8 ± 0.7^*^	1.6 ± 0.2^*,**^
C20:5n3 (cis-5,8,11,14,17-Eicosapentaenoic)	2.2 ± 0.4^*^	1.9 ± 0.1^**^	2.0 ± 0.1^*,**^	1.8 ± 0.0^**^
C22:2 (cis-13,16-Docosadienoic)	1.9 ± 0.4^*^	2.0 ± 0.4^*^	1.7 ± 0.3^*^	1.6 ± 0.1^*^
C22:6n3 (cis-4,7,10,13,16,19-Docosahexaenoic)	3.9 ± 0.6^*^	3.8 ± 0.5^*^	3.0 ± 0.2^**^	2.9 ± 0.1^**^
SFA	59.5 ± 2.6^**^	62.7 ± 2.2^*^	59.8 ± 1.1^**^	59.2 ± 1.1^**^
MUFA	21.8 ± 1.7^**^	21.8 ± 1.0^**^	24.7 ± 0.9^*^	23.2 ± 0.3^*,**^
PUFA	9.6 ± 1.3^*^	9.9 ± 0.7^*^	10.7 ± 1.2^*^	9.6 ± 0.5^*^
PUFA/SFA	0.4 ± 0.0^*^	0.3 ± 0.0^**^	0.4 ± 0.0^*^	0.4 ± 0.0^*^
n-6 PUFA	1.6 ± 0.1^***^	2.2 ± 0.2^***^	4.1 ± 0.9^*^	3.2 ± 0.4^**^
n-3 PUFA	6.2 ± 1.0^*^	5.7 ± 0.5^*,**^	5.0 ± 0.1^**,***^	4.8 ± 0.1^***^
n-6/n-3	0.3 ± 0.0^**^	0.4 ± 0.0^**^	0.8 ± 0.2^*^	0.7 ± 0.1^*^
EPA+DHA	6.2 ± 1.0^*^	5.7 ± 0.5^*,**^	5.0 ± 0.1^**,***^	4.8 ± 0.1^***^

Results are expressed as mean ± standard deviation in percentage (%). A different number of stars in the same column indicates a significant difference (P < 0.05) by Tukey test between different fermentation times in the laboratory.

SFA: total saturated fatty acids. MUFA: total monounsaturated fatty acids. PUFA: total polyunsaturated fatty acids. EPA: eicosapentaenoic acid. DHA: docosahexaenoic acid.

The content of n-6 PUFA increased to different extent with concomitant decrease in the proportion of n-3 PUFA along the time. Hence the ratio of n-6/n-3 PUFA was always significantly increased which was considered as non-desirable for obesity prevention [42]. After six months of fermentation, the sum of eicosapentaenoic acid (EPA) and docosahexaenoic acid (DHA) contents significantly decreased by 23%, indicating that degradation reactions had taken place. n-6 PUFAs are more prone to be released by lipolysis [43]. Free fatty acids (FFAs) are the primary substrates for lipid oxidation and are crucial in the flavour development of fermented fish products. The FFAs content depends on lipolysis, lipid oxidation, and the presence of microorganisms [44]. It can be concluded that the longer the fermentation duration, the lower the EPA and DHA levels, while the n-6/n-3 PUFA ratio increases. However, as the complete lipid profile analysis was done only on lab samples, a complementary analysis of market samples could allow to confirm whether, under real commercial and storage conditions, PUFA are effectively degraded.

### Peroxide values of fish paste at four times of controlled fermentation and from the market

**Fig 2:**
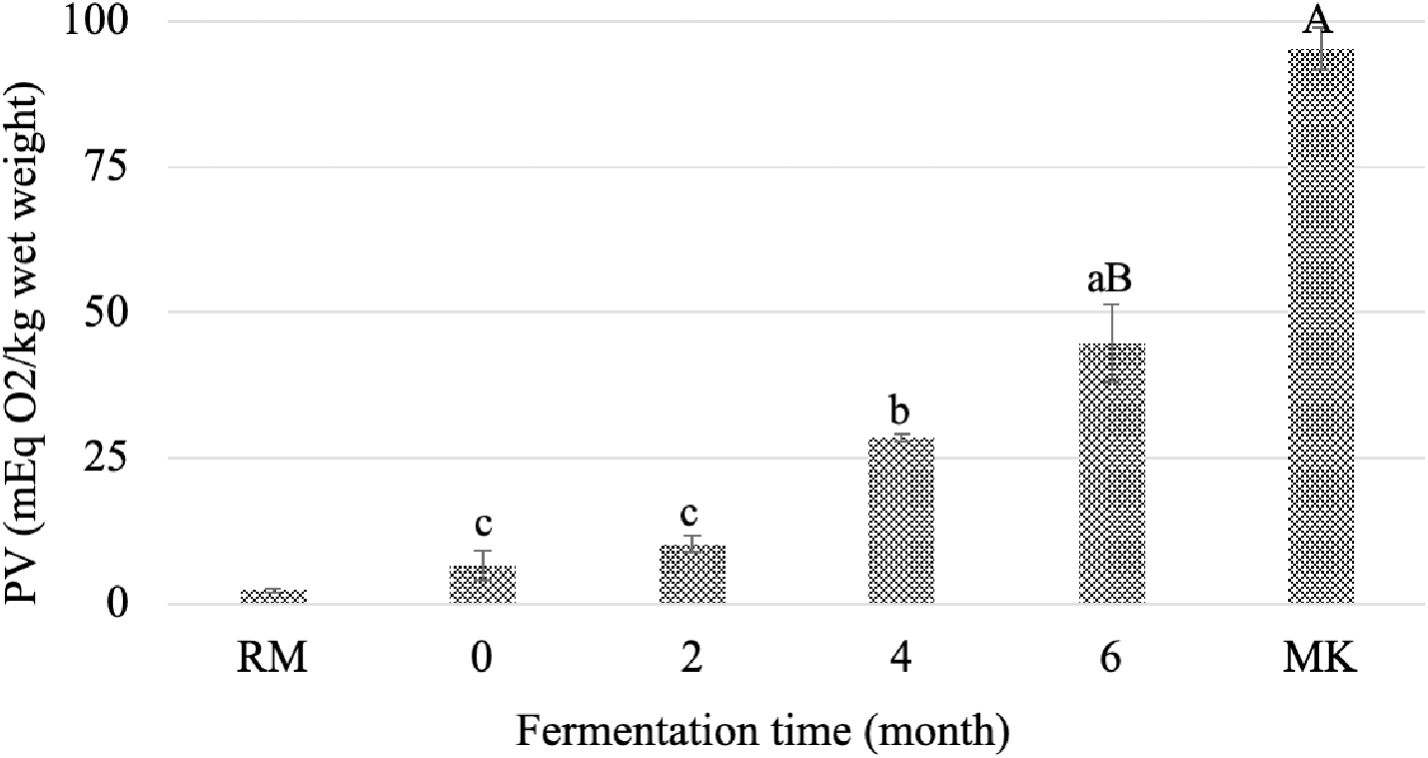
Peroxide values (mEq O_2_/kg wet weight) of fish paste at four times of controlled fermentation and from the market. Results are expressed as mean ± standard deviation on the basis of wet weight. Lowercase letters indicate significant differences (*P*<0.05) between the different fermentation times according to Tukey’s test. Uppercase letters indicate significant differences (P<0.05) between the lab and market samples according to Tukey’s test. RM: Raw material. MK: Market.

The raw material has a low level of PV indicating that the fish used to process the paste were fresh before processing (Fig 2). Lipid peroxidation took place during the first stages of fish paste preparation even if the jars were stored closed in the dark. From the 4th month, the PV exceeded the limit defined for oils in the Codex Alimentarius (10 meq O_2_/kg) [45] and continued to increase until the end of the operation. As a consequence, the lipid profile is altered during past production, with the most unsaturated lipids being degraded first [46]. The products sampled in the local markets have higher PV contents than the lab-produced paste. Storage conditions were probably less controlled, with possible high exposure to light and oxygen.

Oxygen is initially present at the headspace of the bottle, as it is not completely filled with fish paste. This oxygen plays a key role in the initiation of oxidation reactions. The bottles used to prepare the paste under laboratory conditions were made of PET (see the Materials and Methods section). This material was identified during the survey carried out to characterize the recipes in communities. According to the literature, the O_2_ diffusion rate through this material is approximately 3.24 × 10⁻⁸ cm² s^–1^ [47]. The oxygen permeability of packaging depends on its polymer composition, structure and thickness. But, even if the O_2_ diffusion rate is low, oxidation reactions are occurring over a six-month period. Other types of containers (i.e., glass) can also be used during preparation and the dynamic of oxidation reactions could be slightly different.

Peroxides regular consumption can induce oxidative stress, trigger inflammation, and increase the risk of chronic and cardiovascular diseases. Prolonged exposure may also lead to liver toxicity and impair its proper function.

### Total and free amino acid contents of fish paste at four times of controlled fermentation

The TAA and FAA contents of the fish paste during the fermentation were analyzed (Table 3). By the end of the fermentation, glutamic acid was the most abundant AA in the fish paste, followed by lysine and aspartic acid. Glutamic and aspartic acids have also been identified as the predominant AA in other raw fish species, such as herring [48], Atlantic bonito [49], cod [50], and swordfish [51]. Aspartic acid and glutamic acid play an important role as they promote enzyme solubility and ionisation [52]. Lysine is also a crucial AA that the human body needs, and the recommended daily intake for adults is 30 mg/kg of body weight [53].

**Table 3:** The total and free-amino acids of fish paste at different times of controlled fermentation (n = 4).

	Total amino acids (mg/100 g)		Free amino acids (mg/100 g)				
	**RM**	**Month 6**	**RM**	**Month 0**	**Month 2**	**Month 4**	**Month 6**
**Non-essential amino acids**							
Alanine	1171 ± 5^*^	1039 ± 9^**^	20.8 ± 0.6	13.3 ± 2.3^****^	103.3 ± 4.5^***^	130.7 ± 11.7^**^	174.7 ± 4.6^*^
Arginine	1357 ± 7^*^	865 ± 37^**^	ND	ND	ND	ND	ND
Aspartic acid	2299 ± 21^*^	1889 ± 18^**^	ND	2.5 ± 0.6^****^	94.3 ± 9.6^***^	133.1 ± 12.2^**^	186.1 ± 6.5^*^
Cystine	ND	ND	4.2 ± 0.5	5.0 ± 0.7^****^	27.4 ± 1.3^***^	29.3 ± 1.7^**^	41.9 ± 1.8^*^
Glutamic Acid	2138 ± 18^**^	2393 ± 44^*^	11.5 ± 0.2	8.9 ± 2.1^****^	180.3 ± 11.1^***^	225.7 ± 20.6^**^	276.7 ± 3.2^*^
Glycine	883 ± 16^*^	752 ± 17^**^	46.5 ± 1.4	9.7 ± 0.8^****^	28.3 ± 2.0^***^	40.2 ± 3.6^**^	56.4 ± 2.8^*^
Serine	780 ± 20^*^	673 ± 47^**^	7.8 ± 0.3	4.3 ± 1.0^****^	46.0 ± 1.4^***^	61.3 ± 5.2^**^	88.4 ± 4.1^*^
Tyrosine	790 ± 2^*^	637 ± 17^**^	1.0 ± 0.4	1.1 ± 0.6^****^	20.6 ± 0.6^***^	30.8 ± 2.7^**^	45.8 ± 10.6^*^
Proline	820 ± 1^*^	637 ± 45^**^	1.8 ± 0.1	4.6 ± 0.5^****^	73.9 ± 6.0^***^	81.9 ± 5.1^**^	118.4 ± 3.5^*^
**Essential amino acids**							
Histidine	506 ± 1^*^	384 ± 17^**^	13.8 ± 0.6	3.0 ± 1.1^****^	25.1 ± 2.1^***^	32.0 ± 2.6^**^	36.4 ± 2.5^*^
Isoleucine	909 ± 1^*^	730 ± 59^**^	4.0 ± 0.2	2.7 ± 0.7^****^	37.6 ± 1.3^***^	54.4 ± 3.9^**^	64.3 ± 1.1^*^
Leucine	1610 ± 1^*^	1365 ± 37^**^	6.5 ± 0.1	4.8 ± 0.7^***^	122.5 ± 8.6^**^	155.9 ± 17.3^*^	170.7 ± 5.5^*^
Lysine	1806 ± 4^a^	1626 ± 40^**^	11.0 ± 1.1	13.8 ± 1.2^****^	168.5 ± 10.8^***^	206.1 ± 17.9^**^	253.4 ± 8.5^*^
Methionine	672 ± 5^*^	582 ± 19^**^	5.1 ± 0.2	5.8 ± 0.4^****^	47.1 ± 3.3^***^	59.6 ± 4.4^**^	78.8 ± 7.3^*^
Phenylalanine	916 ± 2^*^	706 ± 24^**^	3.2 ± 0.2	3.6 ± 1.0^****^	27.3 ± 2.2^***^	39.7 ± 4.2^**^	48.0 ± 0.9^*^
Threonine	854 ± 22^*^	771 ± 23^**^	11.0 ± 0.2	1.6 ± 0.8^****^	40.5 ± 1.0^***^	58.1 ± 6.2^**^	84.1 ± 1.3^*^
Tryptophan	170 ± 3^**^	185 ± 6^*^	ND	ND	ND	ND	ND
Valine	779 ± 0.3^*^	693 ± 47^**^	5.8 ± 0.2	5.9 ± 0.9^****^	64.3 ± 3.2^***^	82.6 ± 7.7^**^	103.1 ± 1.6^*^
ƩAA	18459 ± 78^*^	15925 ± 296^**^	153.6 ± 3.3	153.2 ± 10^****^	1233.6 ± 69.3^***^	1585.2 ± 128.1^**^	2024.6 ± 48.5^*^
ƩEAA	8222 ± 12^*^	7042 ± 219^**^	60.4 ± 1.7	38.2 ± 3.6^****^	507.7 ± 28.9^***^	656.3 ± 58.2^**^	802.3 ± 20.9^*^
ƩNEAA	10237 ± 90^*^	8883 ± 88^**^	93.2 ± 1.8	52.4 ± 8.0^****^	599.0 ± 26.1^***^	764.9 ± 58.0^**^	1024.6 ± 28.5^*^
ƩEAA/ ƩAA	0.45 ± 0^*^	0.44 ± 0^*^	0.39 ± 0.0	0.42 ± 0.0^**^	0.45 ± 0.0^*^	0.46 ± 0.0^*^	0.43 ± 0.0^**^
ƩEAA/ ƩNEAA	0.80 ± 0^*^	0.79 ± 0^*^	0.65 ± 0.0	0.73 ± 0.0^***^	0.84 ± 0.0^*^	0.85 ± 0.0^*^	0.78 ± 0.0^**^

Results are expressed as mean ± standard deviation per 100 g of wet weight. A different number of stars in the same column indicates a significant difference (P < 0.05) by Tukey test between different fermentation times in the laboratory.

RM: raw material. AA: amino acids. EAA: essential amino acids. NEAA: non-essential amino acids. ND: not detected.

The levels of total amino acids (TAA) significantly decreased after fermentation, while those of free amino acids (FAA) markedly increased. These changes are likely due to the combined effects of salting and enzymatic hydrolysis processes. Indeed, AA are solubilized as salt diffuses through muscle tissues [50]. Additionally, amino acids and carboxyl groups can be released from the ends of protein chains [54]. Salting is known to cause a significant reduction in AA levels [51]. Furthermore, the hydrolysis of proteins into peptides and free amino acids is facilitated by the action of decarboxylase enzymes. These enzymes are either naturally present in the raw material or produced by microbial communities involved in fermentation [55,56].

### Biogenic amine contents of fish paste produced with controlled fermentation and from the market

Ten BAs were identified in the final products (five in the lab samples, and eight in the market samples) (Table 4). The total BA content was significantly higher in samples taken at the market than in those produced in a controlled manner in the laboratory. It can therefore be assumed that the marketed samples are much more contaminated (particularly MK2). In the latter case, total BAI contents were twenty times higher than the acceptable limit of 50 mg/kg [57]. Indeed, values between 50 and 90 mg/kg indicate advanced food degradation.

**Table 4:** Biogenic amine contents (mg/ kg wet weight) in fish pastes from the laboratory and market.

Biogenic amines	Precursors	Lab sample (Month 6)	Market sample (MK1)	Market sample (MK2)
Cadaverine	Lysine	136.8 ± 24.9^**^	147.6 ± 4.0^**^	530.7 ± 3.9^*^
Histamine	Histidine	<LLOQ^***^	51.7 ± 6.8^**^	113.5 ± 9.3^*^
Methylamine	Glycine	8.6 ± 2.8^*^	<LLOQ^**^	<LLOQ^**^
2-phenylethylamine	Phenylalanine	<LLOQ^***^	200.7 ± 5.3^*^	170.4 ± 5.5^**^
Putrescine	Arginine, Ornithine	43.7 ± 4.9^***^	74.6 ± 2.2^**^	149.9 ± 2.5^*^
Serotonin	Hydroxytryptophane	2.2 ± 1.3^*^	2.3 ± 0.1^*^	2.5 ± 0.2^*^
Spermidine	Arginine, Ornithine	<LLOQ^*^	<LLOQ^*^	<LLOQ^*^
Spermine	Arginine, Ornithine	<LLOQ^***^	4.2 ± 0.2^**^	5.5 ± 0.1^*^
Tryptamine	Tryptophan	<LLOQ^***^	18.1 ± 0.5^**^	19.1 ± 0.7^*^
Tyramine	Tyrosine	62.9 ± 11.1^***^	139.9 ± 3.4^**^	207.4 ± 8.2^*^
Total BA		254.6 ± 23.4^***^	639.2 ± 22.5^**^	1199.2 ± 30.0^*^
BAI		243.4 ± 24.0^***^	413.9 ± 16.4^**^	1001.5 ± 23.9^*^

Results are expressed as mean ± standard deviation per kg of wet weight. A different number of stars in the same column indicates a significant difference (P < 0.05) by Tukey test between lab and market samples.

LLOQ: lower limit of quantification. BAI: Biogenic Amines Index=sum of the content of putrescine, cadaverine, tyramine and histamine.

The most abundant BA was cadaverine particularly in the market samples (MK1 and MK2). In the literature, it is reported that the levels of histamine, putrescine and cadaverine can be used to measure the quality of fish and their derived products [28,41]. Histamine is the only biogenic amine regulated by the European Union with a specific threshold (400 mg per kilogram). In a reassuring way, its content in our samples is lower than the legal limit set by the European commission (2073/2005/EC) which indicates that fish pasts should not exceed 200–400 mg/kg [58]. The presence of other biogenic amines is monitored as part of surveillance programs for contamination in fishery products, though no specific limits have been established.

Histidine, arginine and lysine were identified as the precursors of histamine, putrescine and cadaverine respectively. Moreover, it was shown previously that free histidine and lysine are released throughout the fermentation. Some bacteria have the capacity to produce high levels of BA through fermentation or spoilage, particularly during prolonged storage at low temperatures [55]. The production of BA requires the presence of microorganisms with decarboxylase activity and the availability of FAA [59]. Bacteria that have demonstrated a strong ability to produce BA include Enterobacteriaceae (notably *Aeromonas*), *Staphylococcus*, *Lactobacillus*, *Psychrobacter*, *Peptostreptococcus*, *Fusobacterium*, and various Gram-positive bacteria [60].

Histamine is a public health issue for humans, as it can cause poisoning. Biogenic amines, in high doses, are known for their neurological effects, potentially causing headaches, as well as cardiovascular effects, such as palpitations and fluctuations in blood pressure [61]. They can also induce gastrointestinal disorders, leading to nausea, abdominal pain, and diarrhea [61]. At low doses and with chronic exposure, they may increase the sensitivity of the digestive tract, promoting the development of intolerances. Additionally, they could disrupt gut microbiota and increase intestinal permeability.

### Fish digestibility of initial and fermented fish

**Fig 3:**
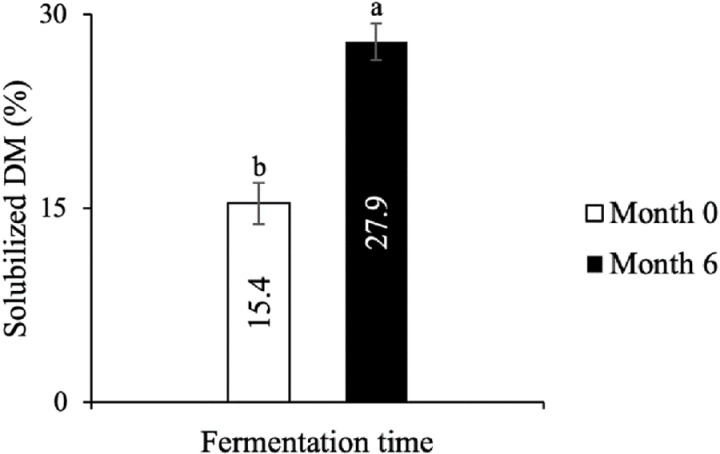
*In vitro* digestibility of initial and fermented fish. Results are expressed as mean ± standard deviation on the basis of wet weight. Different letters indicate a significant difference (*P*<0.05) by Tukey’s test.

The digestibility of raw and fermented fish was estimated using a standard *in vitro* mimicking model (Fig 3). The food matrix becomes significantly more digestible after fermentation, with the solubilized dry matter content nearly doubling (P < 0.05). This result aligns with the observed FAA release throughout the fermentation of fish paste. These FAA are therefore more soluble in biological fluids during *in vitro* digestion. Since the main components of freshwater fish’s dry matter are proteins and lipids [3,4], the solubilized dry matter is likely composed largely of these nutrients, along with salt. Thus, fermentation makes fish more digestible. It can be concluded that this process has the potential to enhance both the speed and efficiency of fish protein digestion.

### Total microflora of fish at different times of controlled fermentation

**Fig 4:**
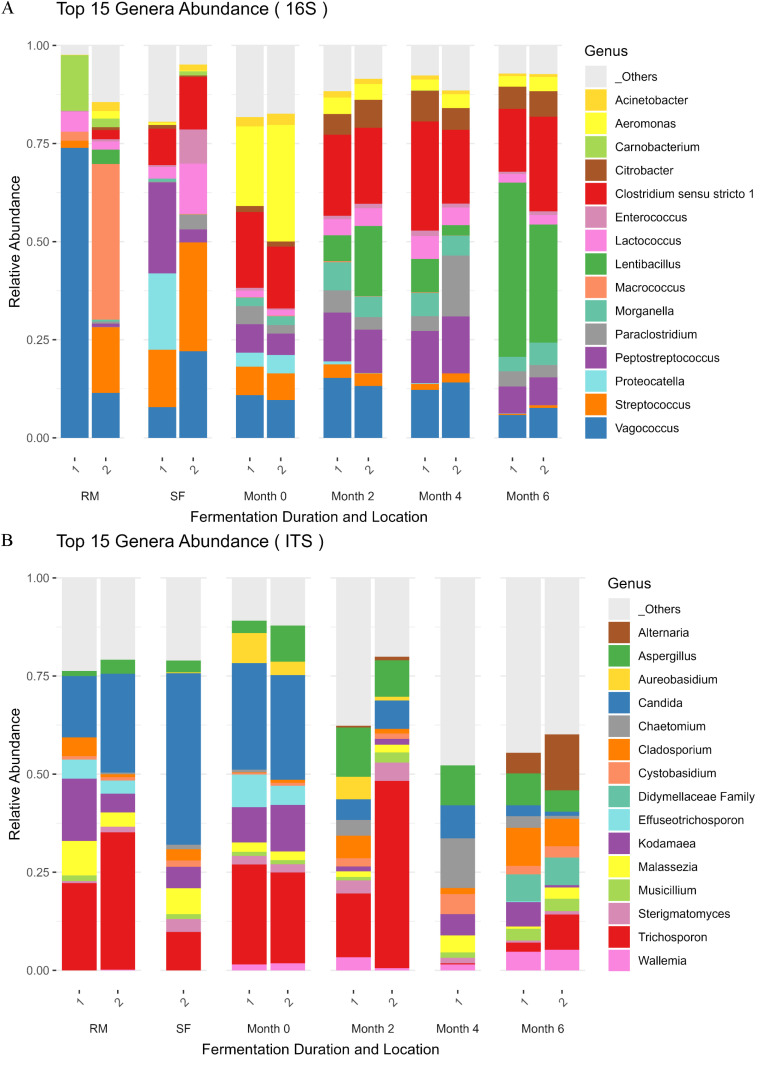
Top 15 genera relative abundance of bacterial (A) and fungal (B) communities of fish in raw material (RM), soaked fish (SF), and at different times of controlled fermentation (month 0, 2, 4 and 6) with three replicates realised in two biological sample repetitions (1, 2). A specific color is assigned to each genus and the correspondence is indicated on the right of the figure.

The plate counting technique showed less than 10 colonies of *Clostridium perfringens* and coliforms and less than 100 colonies of *Bacillus cereus* and *Staphylococcus aureus* in a gram of sample after six months incubation (supplementary material, S1 Table). The use of molecular tools highlighted the bacterial and fungal diversity. A slight difference of the bacterial composition appears between the two biological replicates of raw and soaked fish (Fig 4A). This could be explained by the fact that two different fish fillets were used for the recipe.

In the top 15 genera of bacterial communities, lactic acid bacteria (*i.e., Vagococcus*, *Streptococcus*) are predominant before salting. The genus *Aeromonas* from the *Enterobacteriaceae* family is mostly abundant at the start of incubation (Month 0). *Clostridium* maintains a constant presence over time, whereas the extremely halophilic genus *Lentibacillus* becomes dominant when the product is ready for consumption. *Vagococcus* is known to be widely distributed in aquatic environments, which may explain its occurrence in the early stages of Prahoc production [62]. Similarly, *Streptococcus* has already been detected in the early stages of fermentation [63].

The increase in *Clostridium* could be attributed to its sporulation capacity. This might explain the absence of viable cells observed on the petri dishes, as spores do not readily grow under standard cultivation conditions. The presence of *Clostridium* was confirmed in Prahoc during incubation in another study [14]. The potential strong proteolytic activity of this genus could also explain the increase in FAA during the fermentation (Table 3). This spore-forming genus could pose food safety and consumer health problems. However, it is important to underline that many Clostridium species exist and not all of them are pathogenic species

Several salt-resistant bacteria like Clostridium, Trichosporon, and Candida are capable of producing extracellular proteases, enabling them to efficiently degrade proteins. They specifically break down collagen and release amino acids increasing consequently fish digestibility. Some of these strains could ferment amino acids through Stickland fermentation, producing metabolites such as butyric acid, acetic acid, or biogenic amines (putrescine, cadaverine). Moreover, a previous report noted that another Asian fermented ready-to-eat fish contain *Lentibacillus* [10]. This genus is considered a moderately halophilic bacterial strain; this character trait can explain its resistance to the specifically salty environment of Prahoc [37].

Fungal DNA was difficult to extract and amplify, indicating that the fungal genetic material in our samples may be scarce. At the start of the fermentation, a high abundance of *Candida* yeasts was observed, while *Trichosporon* yeasts were prevalent from the raw material stage up to two months of incubation (Fig 4B). By the end of incubation, fungal diversity was dominated by moulds (*i.e., Alternaria*, *Aspergillus*, *Cladosporium*, *Wallemia)*. The O_2_ diffusion rate through the PET bottles could explain the presence of fungi after six months of fermentation. These fungi have also been found in ready-to-eat fermented fish from Asia and Africa, collected from factories, shops, markets, and e-commerce platforms [10,64].

A summary of the various microbial species presents during the production of Prahoc (supplementary material, S1 Fig). Several of these species have been previously identified (e.g., *Citrobacter freundii*, *Kodamaea ohmeri*, and *Paraclostridium bifermentans*) in fish samples [17,65]. Other species such as *Lentibacillus kimchi*, *Vagococcus teuberi*, *V. penaei*, *V. fluvialis*, *Lactococcus lactis*, and *Trichosporon asahii* have been isolated from a variety of fermented matrices [62,66]. The presence of *T. asteroides*, which exhibits lipolytic activity, may explain the observed increase in n-6 PUFA during Prahoc processing (Table 2) [67]. The *Trichosporon* genus is an opportunistic pathogen that is frequently present in fungal infections that impact persons with weakened immune systems. The pathogenicity of *T. asahii* is extensively studied because of its role in most of trichosporonoses and its increasing resistance to antifungal treatments. Nevertheless, *T. asahii* possesses significant biotechnological potential because of its ability to produce taste compounds and antioxidant molecules. Interestingly, this yeast species also possesses certain potential probiotic uses [68]. Despite the genus *Candida* being often regarded as pathogenic, certain strains of *C. parapsilosis* demonstrate lipase activity, while some strains of *C. metapsilosis* are noted for enhancing flavours [64,69]. Hence, the flavour and unique taste of Prahoc may come from the production of the mentioned yeast species.

**Fig 5:**
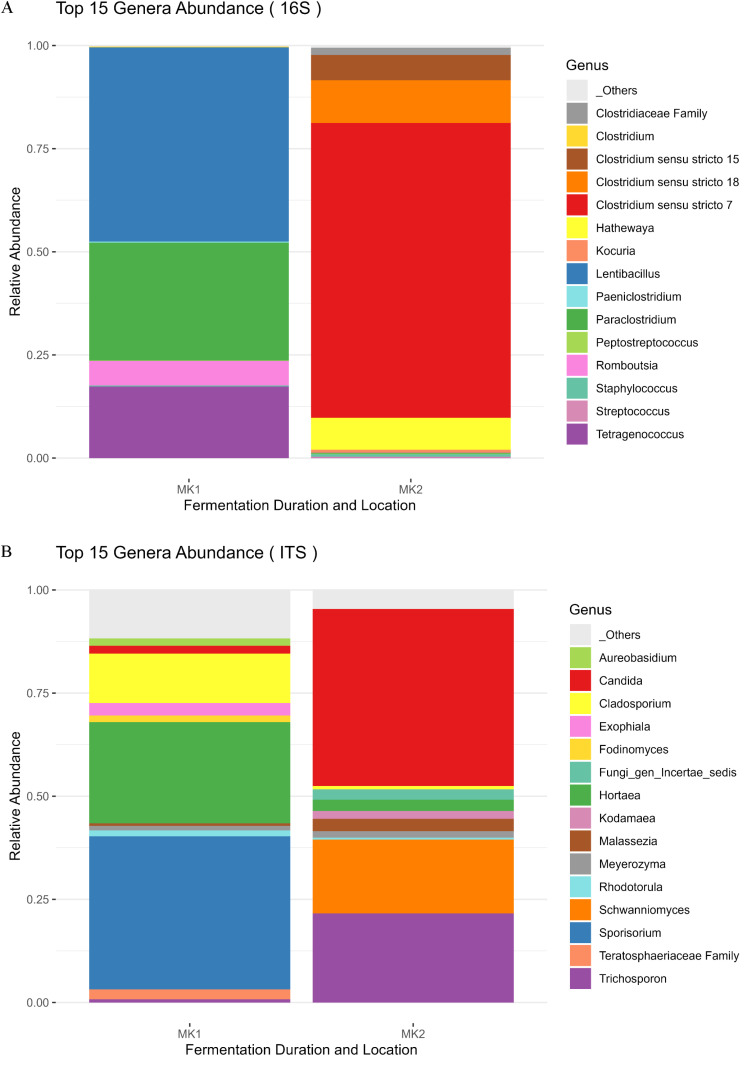
Top 15 genera relative abundance of bacterial (A) and fungal (B) communities in two distinct market samples (MK) analysed once. A specific color is assigned to each genus and the correspondence is indicated on the right of the figure.

*Tetragenococcus* is commonly found in fermented fish according to the literature [14,16], but in our study, it is only detected in MK1 (Fig 5A). The microbial composition of the two market samples differs significantly. The sample market MK1 is predominantly composed of *Lentibacillus* and *Sporisorium*, while MK2 is mainly composed of *Clostridium* and *Candida* (Fig 5B). This could explain the substantial differences in BAI contents between these two samples (Table 4). Previous study found a positive correlation between *Clostridium* and the presence of cadaverine and histamine, and a negative correlation between these compounds and *Lentibacillus* [19].

Prahoc is a traditional product that may contain pathogenic microorganisms (originating from water or spoiled fish), or even parasites. To integrate modern food safety standards in the prahoc traditional recipe, it should use fresh, high-quality fish that is thoroughly washed with clean water (ideally decontaminated by boiling). Fermentation should be done in properly cleaned and airtight containers and the final products should be stored in a clean place, away from light, to prevent undesirable reactions. Before consumption, it should be advised to always cook the paste and not eat it fresh as sometimes done by local communities. To maximize nutritional quality and preserve fragile compounds while minimizing the formation of harmful substances (i.e., peroxides and biogenic amines), it would be advisable to limit the fermentation period to approximately three months.

## Conclusion

The fermentation of Prahoc is a slow process, the duration of which influences its nutritional value, digestibility, and content of potentially harmful compounds (biogenic amines, peroxides). Lipid oxidation intensifies over time, leading to a decrease in essential fatty acids (EPA, DHA). Biogenic amines are produced but remain below the thresholds set by the European Commission.

The main microorganisms involved in the fermentation of Prahoc are salt-resistant bacteria, while fungi remain limited in quantity. The strong proteolytic activity of *Clostridium*, *Trichosporon*, and *Candida* could be responsible for the increase in free amino acids and the product’s digestibility. The presence of *Clostridium*, combined with high concentrations of sodium and peroxides, could pose a significant risk to consumers. This concern is heightened by the essential role of Prahoc in the Cambodian diet, particularly for rural populations, who consume this product more frequently and in larger quantities than their urban counterparts.

This preliminary study highlighted the significant impact of the quality of raw materials, water, and the hygiene conditions of processors on the final quality of Prahoc. It will be important to raise awareness among Cambodian national producers about good hygiene practices to ensure adequate food safety as it was done with the European project Cordis in Asia to improve the knowledge on traditional fish fermentation techniques and improve diffusion of safer practices

## Supporting information

S1 Table Plate counting data after six months incubation. (DOCX)

S1 Fig Heatmaps representing the relative abundance of bacterial (A) and fungal (B) species (taxa represented by at least 1% of total reads) during the preparation of Prahoc at lab scale (n = 3) compared to market samples (n = 1). RM: Raw Material; SF: Soaked Fish.
(DOCX)
