# Supplementary material for: Nutritional and microbiological dynamics in the preparation of prahoc fish paste
Source: PLoS One. 2025 Apr 24;20(4):e0321834. doi: 10.1371/journal.pone.0321834 (PMC12021279; doi:10.1371/journal.pone.0321834)
Supplement: S1 Table — (DOCX) [file pone.0321834.s001.docx]

ND: Not detected. a, b and c: three different Petri Dishes. 1, 2, 3 and 4: Four biological replicates. VRBL: Violet Red Bile Lactose Agar. TSN: Tryptone Sulfite Neomycin Agar.
