# Supplementary figures and images for: Nutritional and microbiological dynamics in the preparation of prahoc fish paste
Source: PLoS One. 2025 Apr 24;20(4):e0321834. doi: 10.1371/journal.pone.0321834 (PMC12021279; doi:10.1371/journal.pone.0321834)

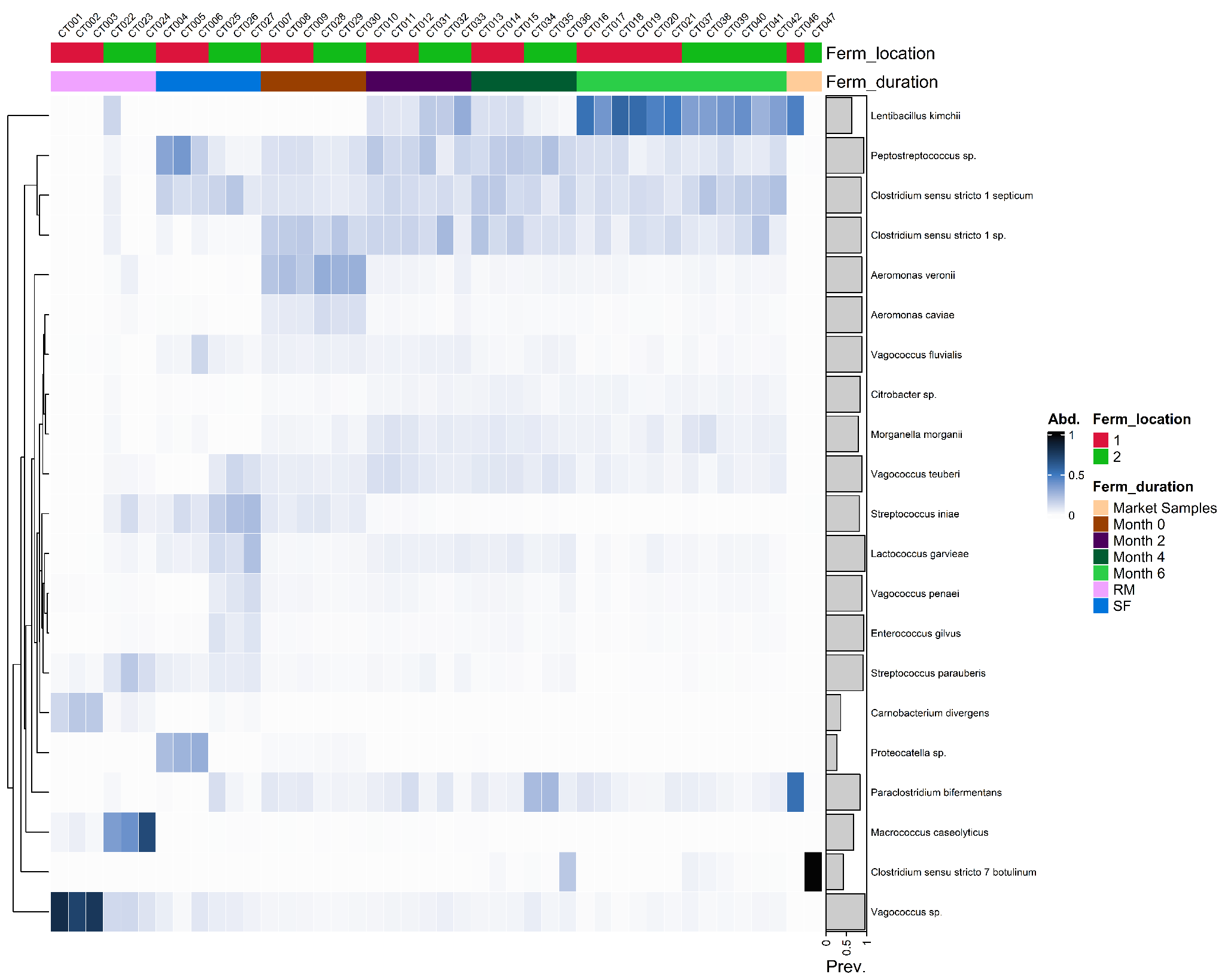


A


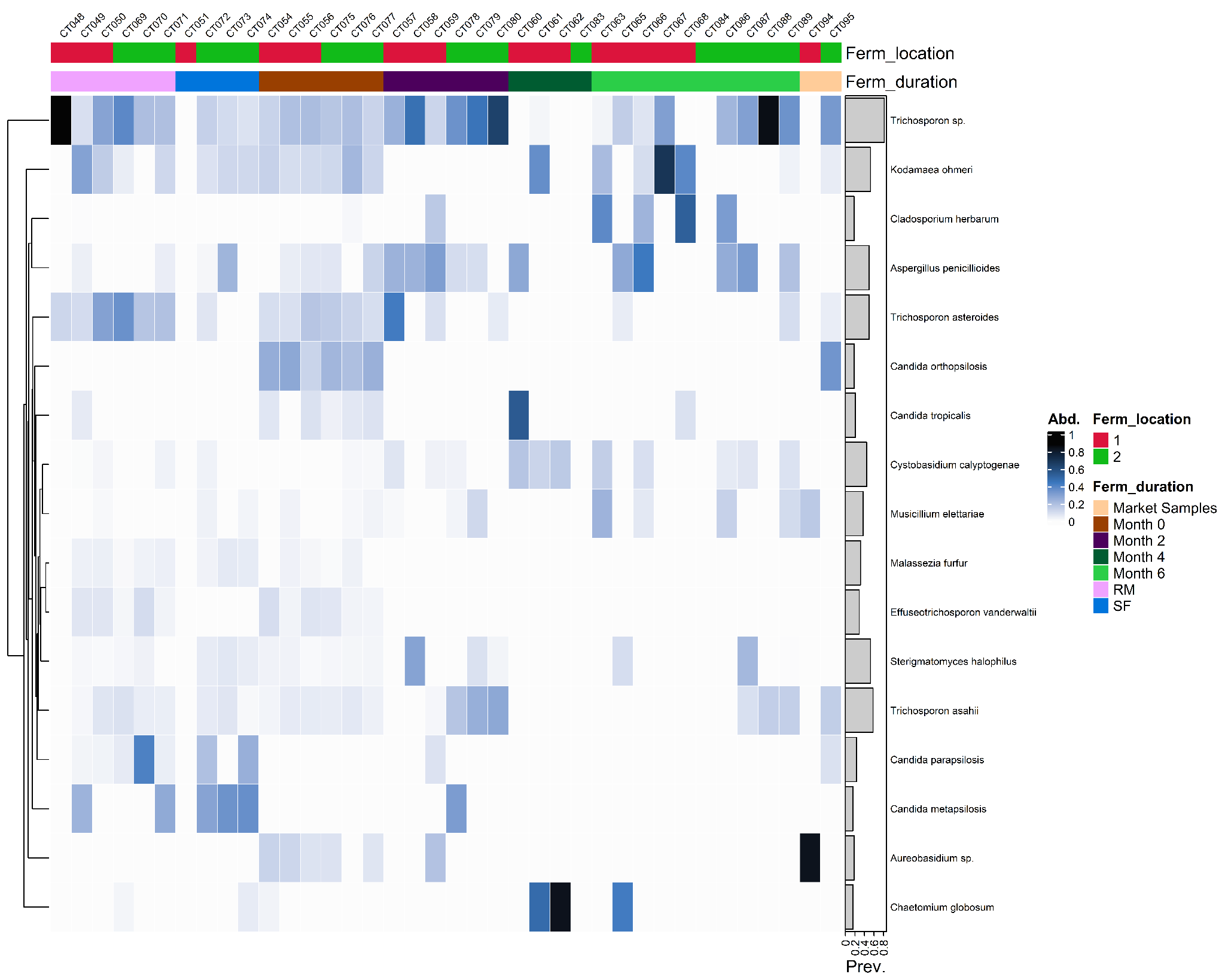


B

Supplement: S1 Fig — RM: Raw Material; SF: Soaked Fish. (DOCX) [file pone.0321834.s002.docx]
